# Weathering a Dynamic Seascape: Influences of Wind and Rain on a Seabird’s Year-Round Activity Budgets

**DOI:** 10.1371/journal.pone.0142623

**Published:** 2015-11-18

**Authors:** Pierre A. Pistorius, Mark A. Hindell, Yann Tremblay, Gavin M. Rishworth

**Affiliations:** 1 DST/NRF Centre of Excellence at the Percy FitzPatrick Institute, Department of Zoology, Nelson Mandela Metropolitan University, Summerstrand 6031, South Africa; 2 Institute for Marine and Antarctic Studies, University of Tasmania, Tasmania 7001, Australia; 3 Institut de Recherche pour le Développement, UMR EME-212 Exploited Marine Ecosystems, Centre de Recherche Halieutique Méditerranéenne et Tropicale, Avenue Jean Monnet BP 171, 34203 Sète cedex, France; INIBIOMA (Universidad Nacional del Comahue-CONICET), ARGENTINA

## Abstract

How animals respond to varying environmental conditions is fundamental to ecology and is a question that has gained impetus due to mounting evidence indicating negative effects of global change on biodiversity. Behavioural plasticity is one mechanism that enables individuals and species to deal with environmental changes, yet for many taxa information on behavioural parameters and their capacity to change are lacking or restricted to certain periods within the annual cycle. This is particularly true for seabirds where year-round behavioural information is intrinsically challenging to acquire due to their reliance on the marine environment where they are difficult to study. Using data from over 13,000 foraging trips throughout the annual cycle, acquired using new-generation automated VHF technology, we described sex-specific, year-round activity budgets in Cape gannets. Using these data we investigated the role of weather (wind and rain) on foraging activity and time allocated to nest attendance. Foraging activity was clearly influenced by wind speed, wind direction and rainfall during and outside the breeding season. Generally, strong wind conditions throughout the year resulted in relatively short foraging trips. Birds spent longer periods foraging when rainfall was moderate. Nest attendance, which was sex-specific outside of the breeding season, was also influenced by meteorological conditions. Large amounts of rainfall (> 2.5 mm per hour) and strong winds (> 13 m s^-1^) resulted in gannets spending shorter amounts of time at their nests. We discuss these findings in terms of life history strategies and implications for the use of seabirds as bio-indicators.

## Introduction

The behavioural and physiological response of animals to varying environmental conditions is a central theme in ecology [1–4]. Due to physiological constraints, animals are limited in the range of environmental conditions they can tolerate, which largely dictates species’ distributional ranges. Within the physiological constraints, behavioural adaptations and flexibility can, however, greatly influence individual fitness, and provide the capacity to deal with and respond to fluctuating conditions [5, 6].

Among animal taxa, seabirds, particularly in temperate and polar latitudes, are among the most challenged by variable and often unpredictable environmental conditions [7, 8]. Flight, as the primary mode of locomotion in most seabirds, as well as prey searching and detection capacities are influenced by weather conditions, particularly wind [6, 9, 10]. It is therefore reasonable to expect foraging behaviour and success, within an environment of patchily distributed prey resources [11], to be affected by prevailing weather conditions [12], and for individuals to demonstrate behavioural methods to alleviate adverse consequences.

Inclement weather can furthermore profoundly influence reproduction and survival in seabirds. This is through either adult mortality, loss of eggs or chicks at the nest [13, 14], or indirectly by inhibiting foraging and chick provisioning capacity [15]. Being able to optimise foraging effort in varying weather conditions would consequently have clear fitness implications.

Time-activity budgets, (*i*.*e*. the time allocated to different functions), are fundamental to behavioural ecology and often proximately related to changes in demographic parameters [16, 17]. In seabirds, time allocation to foraging and nest attendance during the breeding season has been studied in detail. Particularly, the time spent foraging, as an indicator of effort [18], has received much attention as it is reflective of prey availability [19, 20]. However, the influence of weather conditions on seabird behaviour remains poorly studied, with existing studies largely being restricted to the breeding season [21–24].

During the non-breeding season, seabirds have a less constrained energy budget and increased behavioural repertoire. This is nonetheless a crucial time for the birds as they must recoup condition lost during chick rearing and expend energy on moulting. However, few studies have investigated the behaviour, and behavioural responses to specific environmental conditions, outside the breeding season [25, 26]. This is despite the likelihood that the flexibility in behaviour, and the removal of the trade-off between energy allocation to breeding and self-maintenance, allows assessment of relative energetic costs and choices when it comes to dealing with the physical environment. Fine-scale, year-round behavioural data for volant seabirds, to accomplish this, are however lacking.

The Cape gannet (*Morus capensis*), listed as ‘Vulnerable to extinction’, is a Southern African endemic, breeding during the austral summer on just six islands. Recent changes in prey availability have caused major declines in numbers at most of these sites [27]. Cape gannets prey on small shoaling pelagic fish, particularly anchovies (*Engraulis encrasicolus*) and sardines (*Sardinops sagax*), by surface plunge diving [28]. Like most Sulids (excluding Northern [29] and Australasian [30] gannets) and many other seabird species, adults do not migrate to distinct wintering grounds [31, 32] and return to their nesting sites outside the breeding season [33]. Furthermore, they utilise the same feeding habitat year-round [34]. Behaviour of this species outside the breeding season is poorly studied [31], despite this probably being a critical period in terms of their population dynamics [35].

The aim of the study was to document year-round time-activity budgets for Cape gannets and to better understand how weather parameters such as wind and rainfall might affect foraging activity and time allocation to nest attendance throughout the entire annual cycle.

## Materials and Methods

### Ethics statement

Access to the study site (Bird Island) and permission to conduct this study was granted by South African National Parks (SANParks), the managing authority of this island. SANParks Animal Use and Care Committee (AUCC) approved the study. Ethics clearance was issued by the Research Ethics Committee at the Nelson Mandela Metropolitan University (reference: A10-SCI-ZOO-008). Transmitters attached to study birds did not negatively affect chick growth-rates or fledging success, nor adult foraging trip or nest attendance durations [18].

### Study site

This study was conducted at the Cape gannet breeding colony at Bird Island, Algoa Bay, South Africa (33° 50’ S, 26° 17’ E). Bird Island is situated within the transition zone of the Agulhas Current and the Benguela ecosystem [36], 70 km east of Port Elizabeth. Globally, it hosts the largest gannetry at approximately 90,000 breeding pairs [27].

### Data collection

During the 2011/2012 and 2012/2013 breeding seasons, both partners of 20 (10–15 December 2011) and 30 (5–12 December 2012) pairs of adult Cape gannets attending a young chick (mean age at deployment: 22.4 (± 7.1 SD) days old) were fitted with PVC leg-rings to which coded 4.5g VHF transmitters (NTQB-6-2; Lotek Wireless Inc., Newmarket, Ontario, Canada) were attached to continuously monitor their nest attendance [18]. The transmitters sent a coded signal every 39–40 s which was received by a VHF receiver (DataSika-C5; Biotrack Ltd., Dorset, UK) permanently stationed on the island whenever the birds were within the receiver’s range [18]. Two control transmitters placed in the colony were used to continuously validate receiver functionality. Data were downloaded from the receiver on an approximately monthly basis from December 2011 until September 2014 (thereby covering three Cape gannet breeding and non-breeding seasons).

At the time of transmitter deployment a few breast feathers were plucked for subsequent genetic sexing [37]. Prior to deployment, all attended chicks were carefully removed from their nests, weighed (to the nearest 10 g or 25 g) and their culmen (to the nearest mm) and forewing length (to the nearest mm) measured before being returned to their nests. Chick age was calculated from these morphometric measurements following Mullers *et al*. [38]: when forewing length was less than 40 mm, age = −ln((89.78 –*b*/6.15 x *b*)/0.086) + 0.5, and when forewing length was greater than 40 mm, age = 1.395 − ln(ln(588.8/*w*)/0.0264) + 0.5, where *b* is culmen length (in mm) and *w* is forewing length (in mm). When the date of chick hatching could not be back-calculated from chick age (for example, in the following breeding seasons when the equipped birds or their chicks were not disturbed), chick hatching date was determined from breeding season foraging trip durations based on a known, clear adult behavioural distinction between incubation [39] and chick-rearing [17] in Cape gannets. Parental breeding stage was allocated according to chick age: incubation (up to 50 d before hatch date [40]), guard and post-guard (up to 50 d and 100 d following hatching, respectively [17]), and post-fledge (between 100 d and 150 d following hatching) when parents no longer provision for their offspring. The non-breeding season was defined as the end of February until the beginning of August [41].

Meteorological parameters (wind speed (m s^-1^), wind direction and rainfall (mm h^-1^)) were obtained from the permanent South African Weather Service station on Bird Island. The meteorological data were not continuous due to sporadic periods (usually ≤ 1 d) of erroneous parameter recording, as indicated by nonsensical (“9999”) values. Wind speed was expressed as a continuous variable. Prevailing wind direction was grouped into north, north-east, east, south-east, south, south-west, west and north-west.

### Data analysis

Raw data downloaded from the VHF receiver were converted to trip and nest attendance durations at a 10-minute resolution using a purpose-built software interface (Y. Tremblay, unpubl.) designed in MatLab (MathWorks, Natick, MA, USA). This program minimised erroneous gaps in the data arising from environmental or radio noise and omitted any data from non-functional transmitters (see [18]).

All statistical analyses were conducted using R 2.15.1 [42]. Foraging trip and nest attendance durations were right-skewed and therefore these were log-transformed prior to analysis. Each of these were modelled as response variables in separate linear mixed-effects models (LMM; *lmer* in the *lme4* package [43]) fitted by restricted maximum likelihood (REML) for those behavioural bouts initiated during the non-breeding and breeding seasons. Associated meteorological parameters (rainfall, wind speed and wind direction) were averaged for the entire duration of each foraging trip or nest attendance bout. Wind direction was converted from degrees to radians and averaged using *circ*.*mean* in the *CircStats* package [44]. The following predictor variables were allocated for each of the four complete models (breeding and non-breeding season for each of foraging trip and nest attendance bout durations): sex, time of bout initiation, chick age reflected as breeding stage (see above), chick age interacting with sex, month of bout initiation (during the non-breeding season), prevailing wind direction, average wind speed, wind speed interacting with wind direction and average hourly rainfall. Correlation coefficients were calculated for the meteorological parameters to assess possible collinearity. Nest site, individual and season (year-specific breeding or non-breeding season) were specified as random intercepts to account for repeated measures. From each of the four complete models, all permutations of the predictor variables were modelled separately using the *dredge* function in the *MuMIn* package [45] and the Akaike Information Criterion (AIC) score [46] calculated for each. A pseudo-R^2^ was calculated for each of the best-fitting models (lowest AIC scores) to estimate the variance explained by both the fixed- and random-effect predictors [47].

A significance level of α = 0.05 was used and all results are presented as mean ± SE.

## Results

### Year-round foraging trips and nest attendance

A total of 10,722 and 2,436 foraging trips and 10,660 and 2,417 nest attendance bouts were recorded at Bird Island over the study period during the breeding and non-breeding periods respectively (Table 1). The receiver recorded nest attendance behaviour continuously for 1,009 days, barring a period of 8 d in September 2013 when its memory capacity was exceeded.

**Table 1:** Sex-specific monthly patterns of island usage and foraging trips. Shown for adult Cape gannets at Bird Island, Algoa Bay between December 2011 and September 2014.

		*Breeding season*							*Non-breeding season*				
		Aug	Sep	Oct	Nov	Dec	Jan	Feb	Mar	Apr	May	Jun	Jul
*Average number of visits to the nest (per month)*	Male	29.2 ± 4.1	15.5 ± 2.0	14.7 ± 2.2	20.3 ± 2.6	30.2 ± 2.3	31.8 ± 2.7	19.2 ± 2.0	12.4 ± 1.7	4.2 ± 0.7	6.3 ± 1.0	14.3 ± 2.1	20.9 ± 3.7
	Female	29.4 ± 2.9	14.0 ± 1.7	17.1 ± 2.3	21.4 ± 2.2	31.2 ± 2.6	27.3 ± 2.2	15.1 ± 1.6	6.8 ± 1.3	2.5 ± 1.2	5.3 ± 1.0	12.1 ± 3.1	19.7 ± 3.0
*Total number of foraging trips recorded*	Male	730	356	309	406	1511	1369	729	385	89	175	372	544
	Female	793	393	393	470	1529	1173	530	197	15	69	169	452
*Longest foraging trip recorded (d)*	Male	9.9 ± 3.5	4.5 ± 0.4	5.9 ± 0.7	11.0 ± 5.9	7.4 ± 4.2	8.7 ± 3.7	10.7 ± 1.7	12.8 ± 1.7	22.6 ± 3.0	42.5 ± 4.4	33.8 ± 8.4	23.7 ± 6.8
	Female	31.3 ± 10.1	11.7 ± 6.0	6.4 ± 0.8	19.4 ± 12.7	3.2 ± 1.0	5.6 ± 0.6	8.3 ± 0.9	14.1 ± 3.6	39.4 ± 12.2	73.4 ± 4.9	39.1 ± 11.5	76.8 ± 12.5

**Fig 1:**
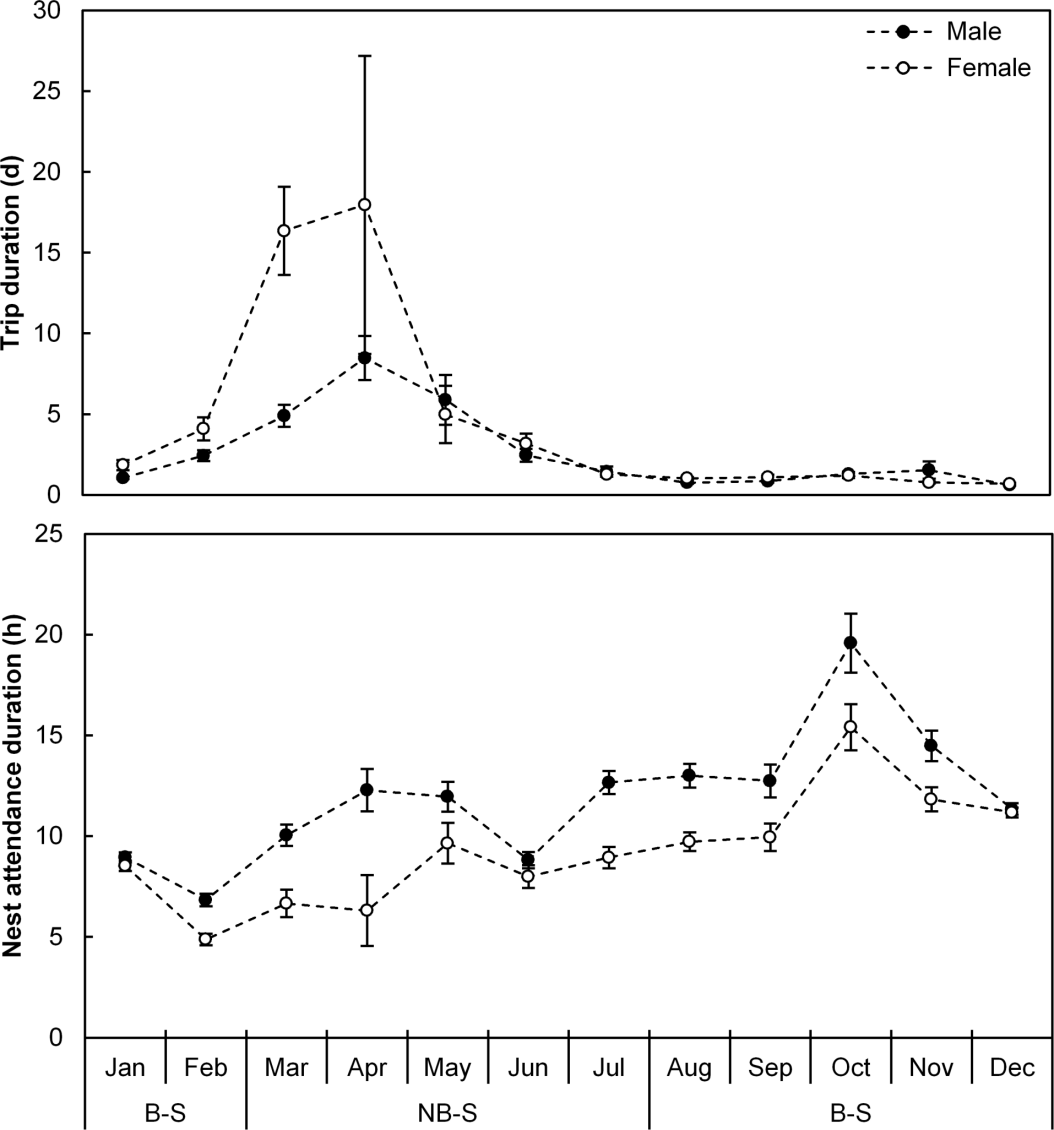
Seasonal Cape gannet time-activity budgets. Trip (top graph) and nest attendance (bottom graph) durations in relation to season for male (dark circles) and female (white circles) adult Cape gannets at Bird Island, Algoa Bay between December 2011 and September 2014. Breeding (B-S) and non-breeding (NB-S) seasons are indicated.

**Fig 2:**
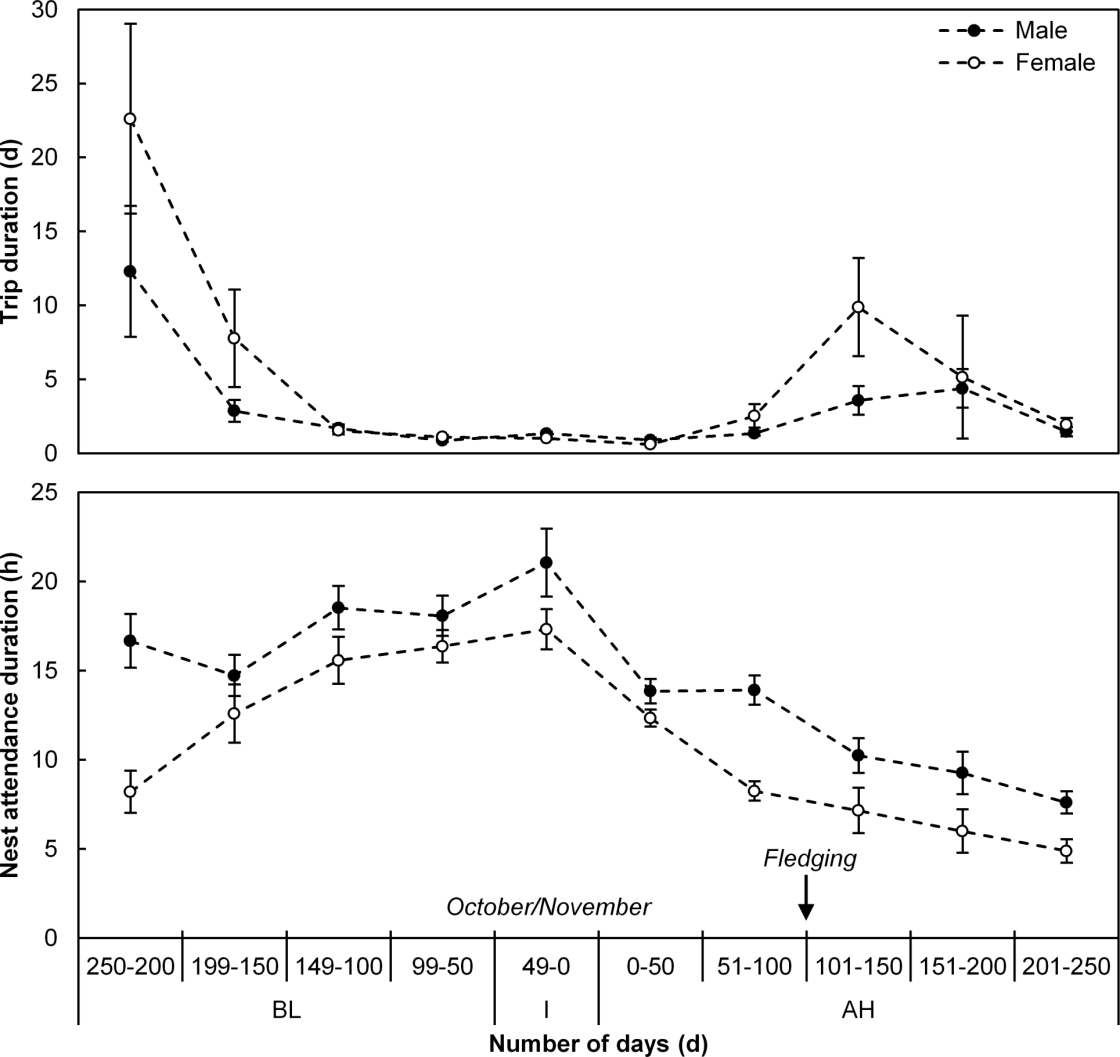
Adult Cape gannet behaviour in relation to chick age. Trip (top graph) and nest attendance (bottom graph) durations in relation to before and after egg hatching (calculated from the automated behavioural data according to published parent activity patterns) for male (dark circles) and female (white circles) adult Cape gannets at Bird Island, Algoa Bay between December 2011 and September 2014. Before laying (BL), incubation (I) and after hatching (AH) periods as well as the peak incubation time of year and the approximate fledging age [17] are indicated.

A distinctive seasonal pattern in duration of foraging trips and nest attendance was apparent (Fig 1). During incubation, the gannets made relatively short foraging trips and had long attendance bouts, on average 27.0 ± 1.9 h and 18.7 ± 1.0 h respectively (Fig 2). Following hatching, a marked decrease in nest attendance duration, associated with brooding shifts, was evident while foraging trips were marginally shorter (Fig 2). Only when chicks neared fledging in February did sex-specific differences become apparent when females made longer foraging trips and spent shorter periods at the nest compared to males (Figs 1 and 2).

During the initial months following the breeding season (March and April), adults tended to embark on long foraging trips (Fig 1). Sex-specific differences persisted with females undertaking foraging trips lasting three times as long as those of males (16.4 ± 2.6 d versus 5.6 ± 0.6 d; Fig 1). Following April, foraging trips were similar in duration between sexes although the longest foraging trips embarked on by each female were longer than those of males (Table 1).

**Fig 3:**
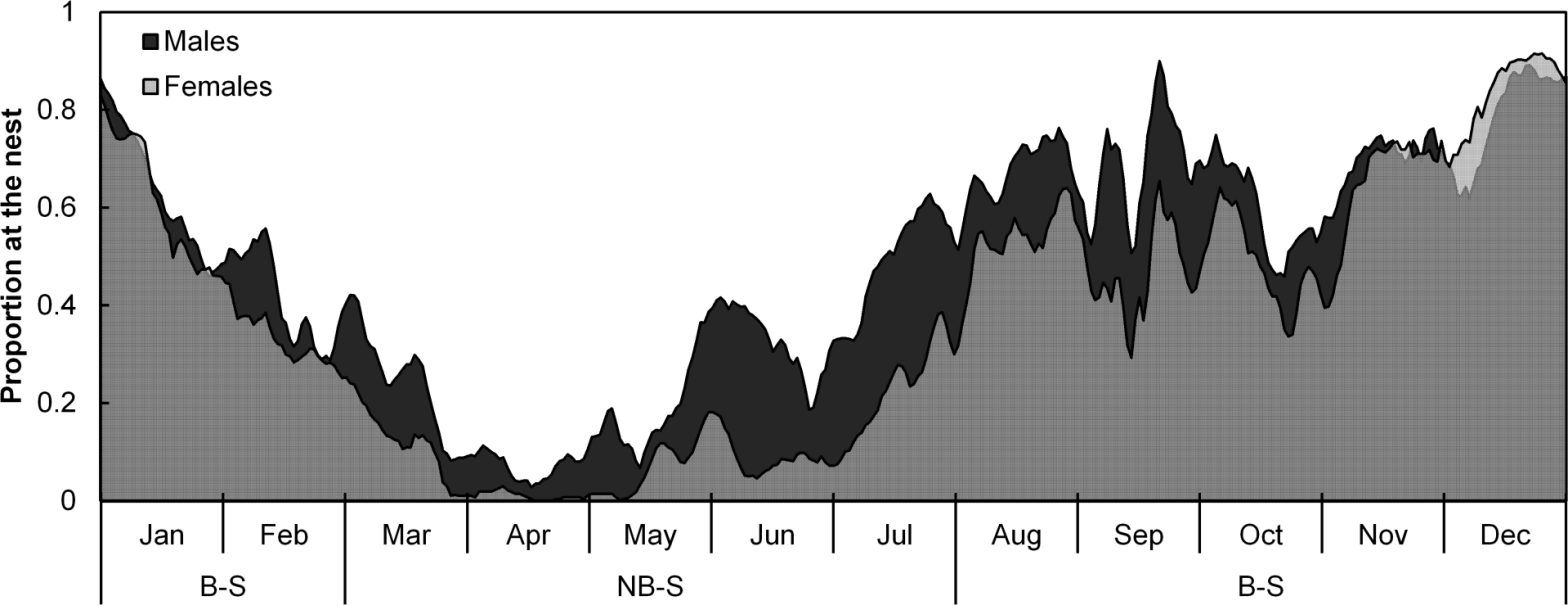
Seasonal patterns of foraging and island usage. Daily proportion (shown as a 7-day moving average) of male (dark shading) and female (light shading) adult Cape gannets with active transmitters at sea or visiting their nests at Bird Island, Algoa Bay between December 2011 and September 2014.

Females consistently spent less time at their nests (Fig 1) and visited the island less often than males (Table 1) soon after the breeding season. In contrast, males tended to maintain a consistent presence at the island during the non-breeding season (Fig 3), visiting their nests on average 4.2 ± 0.7 times per month compared to 2.5 ± 1.2 visits per month by females (Table 1).

From the austral mid-winter onwards, the numbers of both males and females at the island steadily increased, with a larger proportion of males being present on the island throughout this period (Fig 3). Birds progressively spent longer periods at their nests up to a peak in October (Fig 1) which coincided with the period prior to egg hatching (Fig 2).

### Meteorological drivers of behaviour

Due to sporadic periods of incomplete weather data (see Methods), a smaller sample of 11,853 foraging trips (9,550 and 2,303 during the breeding and non-breeding seasons, respectively) and 11,309 nest attendance bouts (9,064 and 2,245 during the breeding and non-breeding seasons, respectively) were used in the models. There was minimal collinearity between the wind and rain data that was used (correlation coefficients were less than 0.065). During the breeding season, foraging trip duration was clearly influenced by breeding stage (increasing with chick age), parental sex and breeding stage interacting with parental sex (Model BT1, Table 2). Rainfall, wind speed and direction and the interaction thereof also influenced foraging trip duration (Model BT1, Table 2). Breeding stage, time of nest arrival as well as all measured meteorological parameters influenced parent nest attendance duration while breeding (Models BN1, Table 2). During the non-breeding season, time of nest departure, month and wind parameters influenced adult foraging trip durations (Models NBT1, Table 2). During this period nest attendance patterns were only influenced by parental sex, month and time of arrival at their nests (Models NBN1 to NBN3, Table 2), although it was marginally affected by rainfall and wind direction (Models NBN3 and NBN4, Table 2).

**Table 2:** Linear mixed-effects models of seasonal Cape gannet behavioural patterns at Bird Island, Algoa Bay. Constructed using log-transformed foraging trip (T) and nest attendance (N) durations as a function of predictor variables during the breeding (August to February) and non-breeding (March to July) seasons: adult sex, time of bout initiation, chick age reflected as breeding stage (see Methods), month of bout initiation, chick age interacting with adult sex, as well as average meteorological parameters over the bout duration which included rainfall, prevailing wind direction, wind speed, and wind speed interacting with direction. Number of parameters for each model (np) and Akaike Information Criterion (AIC) scores, the AIC difference from the most parsimonious model (ΔAIC) as well as the marginal and conditional R^2^ (variance explained by fixed and both fixed and random effects respectively [47]) are indicated. Of the models generated from all permutations of predictor variables, the six with the lowest AIC scores are presented. Black dots and empty spaces indicate variables incorporated or not incorporated in models whereas dashes indicate that the variable was not considered.

Model	Chick age	Sex	Chick age:Sex	Month	Initial time	Rainfall	Wind direction	Wind speed	Wind speed: direction	np	AIC	ΔAIC	R^2^_mar_	R^2^_con_
**Breeding Season**														
*Foraging trip duration*														
BT1	•	•	•	-		•	•	•	•	30	30043.8	0.0	0.068	0.097
BT2	•	•	•	-	•	•	•	•	•	31	30053.0	9.2	0.068	0.098
BT3	•	•	•	-			•	•	•	29	30057.1	13.3	0.066	0.095
BT4	•	•	•	-	•		•	•	•	30	30065.8	22.0	0.066	0.096
BT5	•	•		-		•	•	•	•	26	30084.2	40.3	0.062	0.091
BT6	•			-		•	•	•	•	25	30085.3	41.5	0.060	0.091
*Nest attendance duration*														
BN1	•			-	•	•	•	•	•	26	24612.8	0.0	0.053	0.214
BN2	•	•		-	•	•	•	•	•	27	24617.6	4.8	0.054	0.215
BN3	•			-	•		•	•	•	25	24623.4	10.6	0.052	0.212
BN4	•			-	•	•	•		•	18	24624.1	11.3	0.047	0.200
BN5	•	•		-	•		•	•	•	26	24628.2	15.4	0.052	0.213
BN6	•	•		-	•	•	•		•	19	24629.0	16.2	0.047	0.201
**Non-breeding Season**														
*Foraging trip duration*														
NBT1	-		-	•	•		•	•	•	25	8282.9	0.0	0.122	0.204
NBT2	-		-	•	•	•	•	•	•	26	8285.8	2.9	0.122	0.203
NBT3	-	•	-	•	•		•	•	•	26	8287.8	4.9	0.121	0.208
NBT4	-	•	-	•	•	•	•	•	•	27	8290.7	7.8	0.122	0.206
NBT5	-		-	•			•	•	•	24	8297.0	14.1	0.112	0.197
NBT6	-		-	•		•	•	•	•	25	8299.6	16.7	0.113	0.195
*Nest attendance duration*														
NBN1	-		-	•	•					13	5785.1	0.0	0.037	0.323
NBN2	-	•	-	•	•					14	5786.8	1.7	0.044	0.318
NBN3	-		-	•	•		•			20	5786.9	1.9	0.047	0.334
NBN4	-		-	•	•	•	•			21	5787.1	2.1	0.049	0.332
NBN5	-		-	•	•	•				14	5788.4	3.3	0.038	0.322
NBN6	-	•	-	•	•		•			21	5788.6	3.5	0.054	0.328

**Fig 4:**
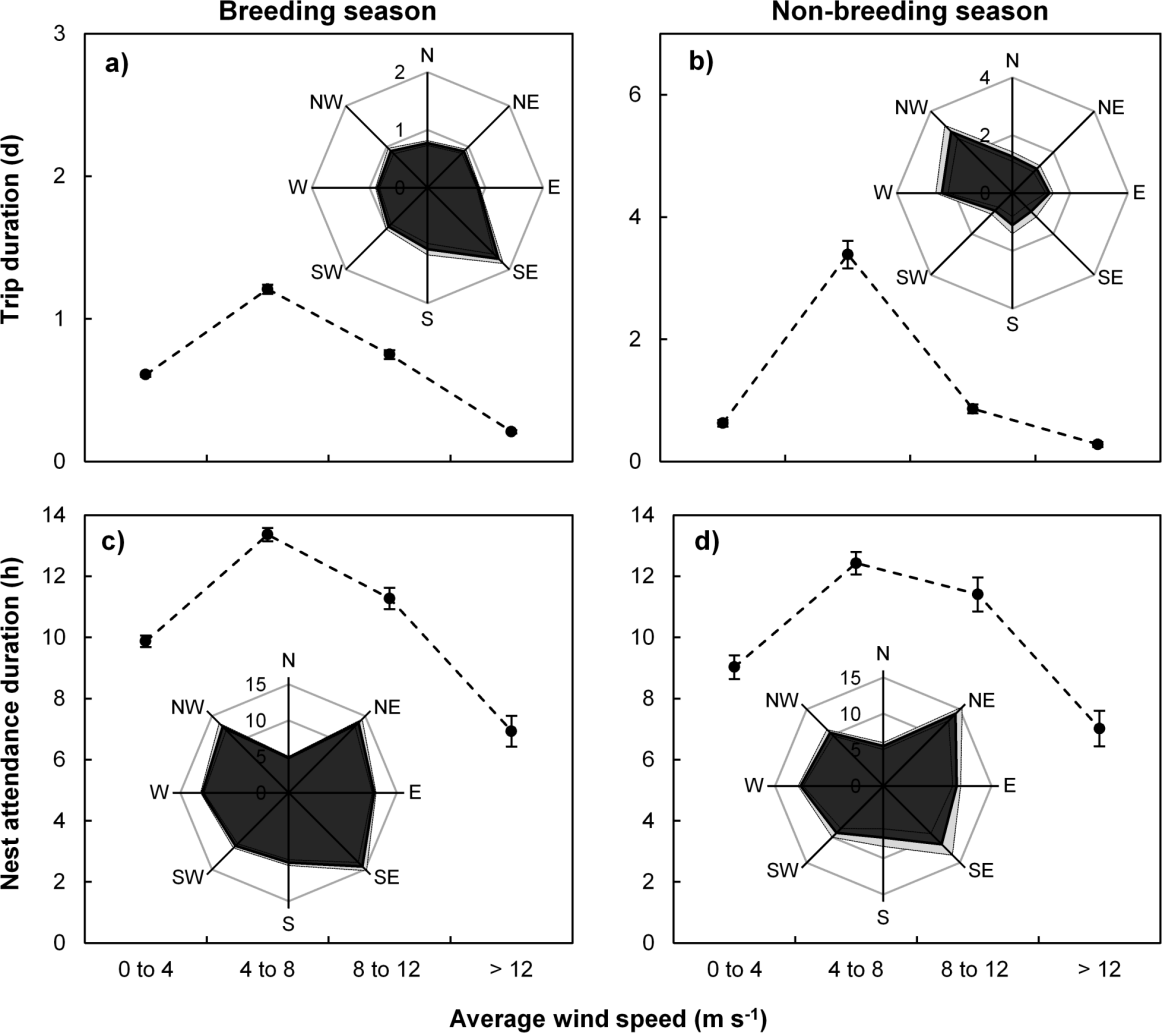
Adult Cape gannet behaviour in relation to wind. Foraging trip (a, b) and nest attendance (c, d) bout durations in relation to wind parameters for Cape gannets at Bird Island, Algoa Bay during the breeding (a, c; August to February) and non-breeding (b, d; March to July) seasons. Bout durations in relation to average wind speed are indicated by the black circles (± SE) connected by a dotted line and in relation to wind direction (N = north, E = east, S = south, W = west) in the inset diagrams (SE estimates are indicated by the light grey shading and inner line in the dark grey).

**Fig 5:**
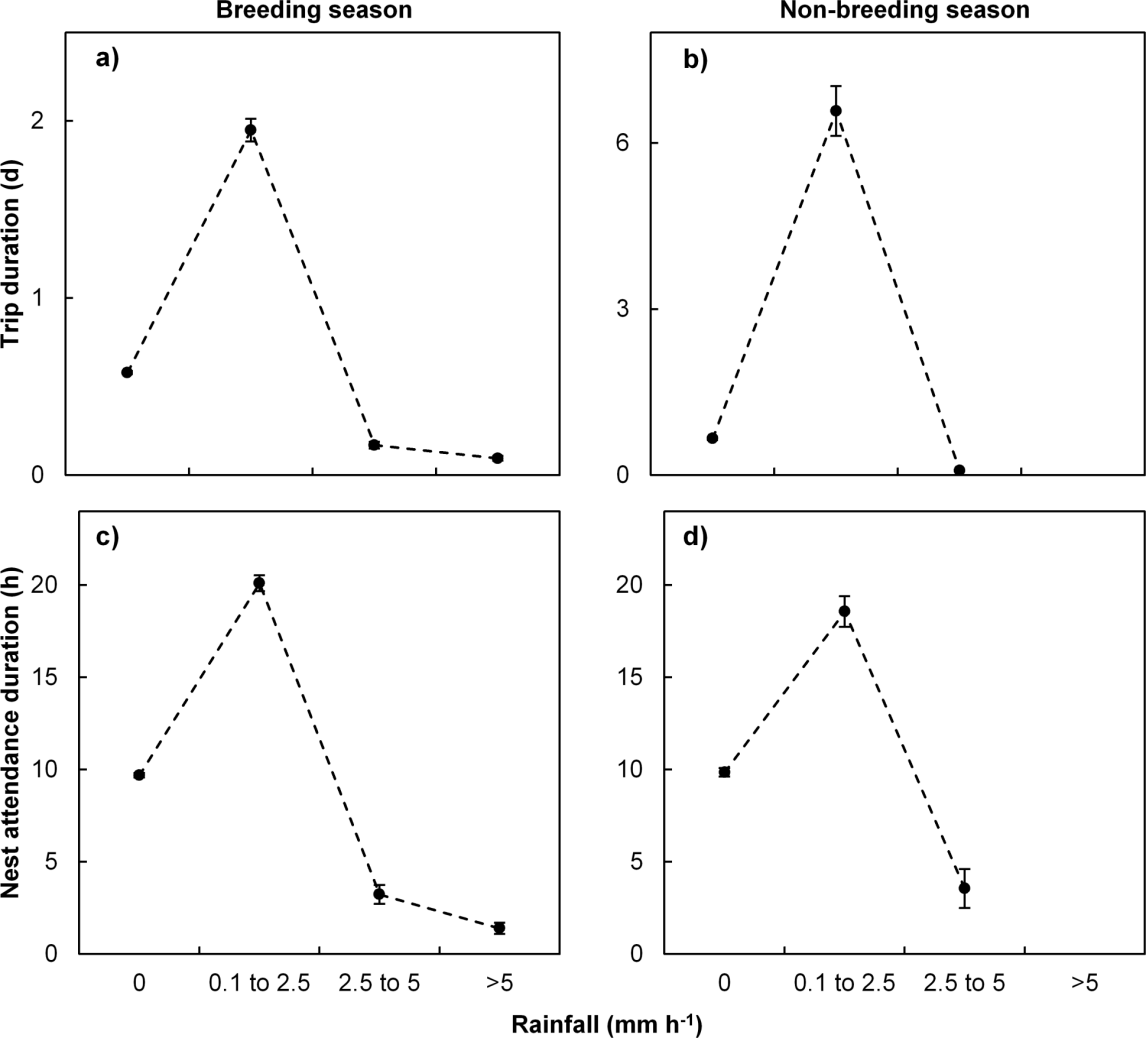
Adult Cape gannet behaviour in relation to rainfall. Foraging trip (a, b) and nest attendance (c, d) bout durations of adult Cape gannets at Bird Island, Algoa Bay in relation to hourly rainfall during the breeding (a, c; August to February) and non-breeding (b, d; March to July) seasons.

Wind direction affected foraging trip duration during the breeding season (Table 2) such that birds experiencing prevailing south-easterly winds while away from the island had relatively long foraging trips on average (Fig 4a). This was opposed to the non-breeding seasons when westerly winds resulted in longer foraging trips (Fig 4b). Furthermore, birds experiencing stronger winds spent shorter periods away from their nests, while during moderate wind speeds (4 to 12 m.s^-1^) birds tended to have extended foraging trip durations (Fig 4a and 4b). Interestingly, birds spent longer periods foraging when experiencing moderate rainfall than during little to no or high rainfall (Fig 5a and 5b). Nest attendance durations were influenced by changes in all measured meteorological conditions during the breeding season in addition to breeding stage and nest arrival time (Table 2). Large amounts of rainfall (> 2.5 mm per hour; Fig 5c and 5d) resulted in shorter amounts of time spent at their nests throughout the year (Model BN1 and NBN4, Table 2).

## Discussion

Using data from over 13,000 foraging trips, providing a continuous, fine-scale assessment of year-round time-activity budgets in a volant seabird, clearly defined annual patterns of island usage and foraging trip durations were evident. Birds attended their nests outside the breeding season with surprising regularity, except for the first 2–3 months following breeding, when extended foraging trips were evident, particularly in females. As reported before, chick developmental stage influenced parental behaviour [17], with a clear transition in behaviour following egg-hatching in the current study. Wind and rain were influential in governing the behaviour of Cape gannets, during or after breeding, or during the entire year.

### Year-round time activity budgets

During the breeding season the energetic demands of offspring and their need for protection largely shapes the activity budgets of adult seabirds [3, 17, 48]. In general, the duration of foraging trips increases and the amount of time spent at the nest decreases through chick development until fledging, with this being particularly apparent after chick-guard when both parents forage simultaneously ([17], this study). Behaviour in seabirds during incubation, during which seabirds with bi-parental incubation strategies alternate incubation shifts with self-replenishment foraging, has also received considerable attention [3, 49, 50]. As found elsewhere [50], the birds spent substantially longer periods of time at the nest while incubating compared to provisioning. This is in all likelihood associated with differing energy budgets. Following hatching, increased foraging time is required in order to provide for offspring and the associated increase in their own energy requirements [51].

Following breeding, Cape gannets, particularly females, embarked on relatively long foraging trips during March and April. Foraging distribution in Cape gannets outside the breeding season has yet to be studied, and it is consequently unknown whether this entails an extension in foraging range, which may be beneficial after local prey resource depletion during the breeding season [52], or whether it simply entails longer foraging trips within the same geographic range. There was substantial variability in trip durations in females during this period (Fig 1). This requires further investigation but could indicate different strategies, with some individuals possibly remaining relatively close to their breeding location while others undertaking more distant foraging trips. The large difference in foraging trip duration between the sexes during this period is also interesting with relatively short foraging trips, and increased nest attendance, possibly being related to greater year-round nest defence responsibilities in males. Following April, sex differentiation in foraging trip durations was not evident (Fig 1), suggesting similar foraging ranges between the sexes during this period.

A high level of breeding ground fidelity during winter was apparent. Although poorly documented and understood in seabirds, remaining close to the breeding colony during winter may be advantageous for a number of reasons. Local knowledge of prey resources may increase foraging efficiency during the austral winter, rather than dispersing to habitats where resources may not be all that predictable [53, 54]. This is particularly relevant in seabirds, such as Cape gannets, that target prey that are spatially dynamic [11]. Maintaining social cohesion outside the breeding season may also be important for foraging during the breeding season with gannets making use of conspecifics for prey location [55–57]. Maintaining and developing pair bonds through regular visits to the nest could furthermore have reproductive benefits and allow for development of synchronised feeding and incubation or provisioning shifts during the breeding season. Returning to land to rest and reduce metabolic rate, which is probably lower on land than when sitting on water [23], may also explain regular nest attendance throughout the year.

Male Cape gannets have been shown to spend more time than females at their nests while breeding [17]. A greater investment in nest attendance by males during the onset of the breeding season and pre-laying period could potentially be attributed to mate-guard [58, 59]. However, from the current study it is clear that this pattern persists throughout most of the year. Although some form of resource partitioning could be driving this, it seems more likely that this difference is socially mediated. Male gannets defend territories against intruders during the breeding season [60], and perhaps to some extent this role continues outside of the breeding season and aids in continued site ownership [31] that carries over into the next breeding season.

### Meteorological influences on behaviour

As expected, meteorological variables influenced bird behaviour and did so differently during and outside of the breeding season. Breeding birds are restricted in their foraging distribution in that they have to return regularly to their nests for incubation, chick provisioning or nest defence. Despite some behavioural phenotypic plasticity, the energetic demands associated with self-maintenance and chick provisioning would imply limited flexibility in response to weather conditions compared to the non-breeding season.

The observed behavioural responses could to some extent have been mediated through weather-driven changes in prey distribution (and availability). There is, however, a dearth of information on the occurrence of such short-term changes in the prey of Cape gannets. We consequently focused our below discussion on the influences of weather parameters on seabird locomotion and prey detection, bearing in mind that prey characteristics (i.e. distributional patterns) could to some extent have influenced observed time-activity budgets. Due to the nature of our data we were unable to independently investigate the influence of weather on flight versus diving behaviour. As shown elsewhere for a volant seabird [61], flight is probably influenced to a greater extent, particularly by wind, but the plunge diving foraging strategy adopted by gannets means that disruption to the water surface (e.g. decreasing visibility during rainfall [62]) could also very possibly influence dive behaviour and success.

Both wind speed and direction as well as the interaction thereof were important predictors of time spent on foraging trips, during and outside of the breeding season (Table 2; Fig 4). Mild to no wind (< 4 m.s^-1^), as well as strong winds, while away from the nest resulted in relatively short foraging trips (Fig 4a and 4b). This would indicate such conditions to be beneficial in terms of either reaching suitable foraging grounds or prey capture. As visual predators, prey capture could possibly be enhanced during calm conditions when birds can readily hone in on target prey specimens without a disruption to the water surface layer. However, should this be the case, then short foraging trips during strong wind conditions require explanation. Perhaps under strong wind conditions prey patch location is enhanced due to wind-supported flight, albeit associated with relatively low rates of prey capture.

Time spent at the nest was influenced by wind speed during the breeding (Fig 4c; Model BN1, Table 2) but not the non-breeding (Fig 4d; Models NBN1 to NBN3, Table 2) season. This could be a reflection of increased discretionary time outside the breeding season allowing birds to spend less time at the island while going on long and possibly distant foraging trips aided by the wind for locomotion. Strong winds have previously been associated with both relatively short [63] as well as long nest attendance bouts in other seabird species, the latter arguably due to poor associated foraging conditions [3, 22].

Wind direction largely influences flight energetics in seabirds and also dictates whether birds can reach suitable foraging grounds [21, 22, 64]. In our study, wind direction influenced foraging duration throughout the year, with south-easterly or westerly winds generally resulting in longer foraging trips during the breeding and non-breeding season respectively (Fig 4a and 4b). Cape gannets from our study colony mostly forage to the south west of the island during the breeding season, in response to larger sardine and anchovy biomass [11]. During the non-breeding season their foraging distribution is not fully known, although there is some evidence that Cape gannets track the sardine run up the east coast during winter [65], when frontal systems and south-westerly winds are frequent. Individuals probably also repeatedly target the same foraging grounds [66]. This would mean that birds would often be flying with the prevailing south-easterly tail winds during the breeding season on their outbound foraging trips [6, 67, 68].

Assuming constant wind conditions, optimal foraging theory would dictate that birds should fly out against the wind, foraging at slower speed which would facilitate prey detection [10], and more efficiently taking off against the wind (which is energetically expensive [23]), to then return to the colony with tailwinds to aid return with additional weight from prey carried to offspring at the colony. However, the dynamic wind conditions that often occurred within the duration of foraging trips mean that birds potentially head out and return against the wind, which would increase transit times and total foraging trip duration. Several studies, including one on northern gannets [68], have demonstrated that seabirds often depart on foraging trips with tailwinds, supposedly due to uncertainty about future wind fields. This is confirmed in the current study, given that the predominant foraging area for Cape gannets is towards the south-west [11]. Another possibility is that the indirect effect of wind outweighs the energetic costs of flights. Upwelling events in Algoa Bay are driven through north-easterly winds [36] and could result in an increase in prey availability, resulting in increased foraging efficiency during these conditions.

Although rain has been associated with seabird behaviour, largely in terms of breeding success and often through large-scale processes such as El Niño southern oscillation [22, 69], to our knowledge this is the first study demonstrating the effect of rainfall on seabird activity budgets. Rainfall has, however, been shown to indirectly influence the foraging behaviour in an inshore foraging seabird through influencing run-off into coastal waters [62]. In our study, moderate rainfall resulted in extended foraging trips which could possibly be due to increased water surface turbidity resulting in poor foraging conditions. It is, however, interesting that shorter observed trips were associated with increasing wind strength, which would also influence the sea surface layer, but perhaps not to the same extent as rainfall does.

Adults spent considerably less time at their nests during the breeding season when it was raining heavily. This is counter-intuitive as increased chick protection would be expected under these conditions, although heavy rainfall could potentially alleviate predation pressure by kelp gulls (*Larus dominicanus*). High breeding failure in the study population has previously been ascribed to prolonged periods of rainfall [14]. From the current study it seems likely that during extended poor weather conditions adults prioritise their own survival over that of their chicks, as would be expected for long-lived species within the framework of life-history theory [70].

### Implications for monitoring

Seabirds are regarded as important sentinels of change and indicators of prey resource availability in marine systems. The premise is that aspects of their biology or behaviour respond in a predictable manner to changes in prey availability (*e*.*g*. [20]). The duration of foraging trips in breeding seabirds are particularly useful to indicate short-term changes in prey availability [19]. While the distribution and behaviour of seabirds, in terms of foraging effort, is often linked to prey biomass and availability [71, 72], physical features of the environment can disrupt this relationship. For example, flight efficiency is largely influenced by wind conditions [9] whereas water clarity and sea surface characteristics could at least in some species influence prey location [73]. As supported by our results, the role of physical events in patterning foraging effort therefore needs to be considered over and above the features of the water masses, which has been studied extensively in the past (*e*.*g*. [7]). Our results indicate behavioural responses in a seabird to varying weather conditions in terms of foraging trip durations. However, only between 9.7 and 33.4% of the variance in foraging trip and nest attendance durations were explained by the combination of explanatory variables used in this study (Table 2). Although prey availability and individual experience probably accounts for most of the remaining variance, these abiotic influences ideally need to be incorporated when relating seabird behavioural responses to prey biomass.
